# Maternal health interventions in resource limited countries: a systematic review of packages, impacts and factors for change

**DOI:** 10.1186/1471-2393-11-30

**Published:** 2011-04-17

**Authors:** Angelo S Nyamtema, David P Urassa, Jos van Roosmalen

**Affiliations:** 1 Tanzanian Training Centre for International Health, Ifakara, Tanzania; 2 Department of Community Health, School of Public Health and Social Sciences, Dar es Salaam, Tanzania; 3 Department of Obstetrics, Leiden University Medical Centre, The Netherlands; 4 Department of Medical Humanities, EMGO Institute for Health and Care Research, VU Medical Centre, Amsterdam, The Netherlands

## Abstract

**Background:**

The burden of maternal mortality in resource limited countries is still huge despite being at the top of the global public health agenda for over the last 20 years. We systematically reviewed the impacts of interventions on maternal health and factors for change in these countries.

**Methods:**

A systematic review was carried out using the guidelines for Preferred Reporting Items for Systematic Reviews and Meta-Analyses (PRISMA). Articles published in the English language reporting on implementation of interventions, their impacts and underlying factors for maternal health in resource limited countries in the past 23 years were searched from PubMed, Popline, African Index Medicus, internet sources including reproductive health gateway and Google, hand-searching, reference lists and grey literature.

**Results:**

Out of a total of 5084 articles resulting from the search only 58 qualified for systematic review. Programs integrating multiple interventions were more likely to have significant positive impacts on maternal outcomes. Training in emergency obstetric care (EmOC), placement of care providers, refurbishment of existing health facility infrastructure and improved supply of drugs, consumables and equipment for obstetric care were the most frequent interventions integrated in 52% - 65% of all 54 reviewed programs. Statistically significant reduction of maternal mortality ratio and case fatality rate were reported in 55% and 40% of the programs respectively. Births in EmOC facilities and caesarean section rates increased significantly in 71% - 75% of programs using these indicators. Insufficient implementation of evidence-based interventions in resources limited countries was closely linked to a lack of national resources, leadership skills and end-users factors.

**Conclusions:**

This article presents a list of evidenced-based packages of interventions for maternal health, their impacts and factors for change in resource limited countries. It indicates that no single magic bullet intervention exists for reduction of maternal mortality and that all interventional programs should be integrated in order to bring significant changes. State leaders and key actors in the health sectors in these countries and the international community are proposed to translate the lessons learnt into actions and intensify efforts in order to achieve the goals set for maternal health.

## Background

Reducing maternal mortality has been at the top of the global health agenda for over the last 20 years and we know that 74-98% of maternal deaths can be averted even in the circumstances of most low income countries [1,2]. The burden, however, is still huge and every year 0.36 million maternal deaths [3], 4 million stillbirths and 3 million early neonatal deaths are related to complications of pregnancy and childbirth globally. This figure is by far higher than the total of 5 million estimated deaths due to HIV/AIDS, tuberculosis and malaria combined [4]. The vast majority (99%) of estimated global maternal mortality occurs in resource limited settings, Sub Saharan Africa accounting for more than half [3,5]. While other regions like Latin America and the Caribbean, and Northern Africa had remarkably reduced maternal mortality ratio (MMR) by 41% and 59% respectively between 1990 and 2008, Sub Saharan Africa had only reduced it by 26%. The annual decrease of maternal mortality in Sub Saharan Africa was 1.7% which is far below the 5.5% annual decline rate, which is necessary to achieve the fifth Millennium Development Goal concerning maternal mortality reduction with three quarters [3]. The existing disparities of the trends in reducing maternal mortality in resource limited countries raise questions about the existing factors for change for replication of evidence-based interventions.

Maternal mortality is a complex problem requiring complex interventions. This article attempts to explore the available evidences, integration of maternal health interventions and the factors influencing implementation in resource limited countries. It challenges the key actors in resource limited countries to acknowledge the problem and scale up the means and advocated measures to address this scandal.

## Methods

### Search strategy description

This review used the guidelines for Preferred Reporting Items for Systematic Reviews and Meta-Analyses (PRISMA) [6]. A comprehensive literature search for relevant articles was carried out from PubMed, Popline, African Index Medicus, general internet sources including reproductive health gateway and Google, hand-searching, reference lists and the grey literature. The following free terms were used for electronic searching: "maternal", "mortality", "interventions", "randomized controlled trials" and "emergency obstetric care indicators". With the help of information resource specialist the search details were designed according to specifications of each database (Additional file 1). The last search was carried out on June 30^th ^2010.

### Inclusion criteria

We included randomized controlled trials (RCTs) and quasi-experimental designs with and those without control groups undertaken in resource limited countries that reported in English language the implementation of interventions, their impacts for maternal health within the past 23 years (i.e. from 1987 after launching the global Safe Motherhood Initiative in Nairobi, Kenya). The decision to start from 1987 was arbitrary and based on the need of reviewing recent data. Although the best and most objective way would have been to assess the impacts of these interventions by using RCTs, the investigators wanted also to review the evidences from quasi-experimental studies. By resource limited countries we meant low income (gross national income [GNI] per capita of $975 or less) and lower middle income countries (GNI $976 - $3,855) as classified in 2008 by The World Bank [7]. We included in the analysis only articles that clearly specified types of interventions, duration of implementation and used either MMR or at least one of the emergency obstetric care (EmOC) services indicators as outcome measures. We also extracted the underlying factors for maternal health interventions from the reviewed articles. These included factors which affected the implementation of interventions and all underlying reasons for insufficient implementation of maternal health interventions in these countries. Insufficient implementation was defined as poor coverage in terms of degree of integration of interventions and area as well as the efficiency of implementation. The investigators were interested in these factors because they could have affected the impacts of the interventions in these countries.

### Exclusion criteria

We excluded articles reporting drug and procedural interventions addressing unique communities (like refugees) and individual medical conditions during pregnancy like malaria, anaemia and pre-eclampsia. Articles whose lists of interventions or implementation period were not clear, success reports over 23 years ago and those from countries other than low income and lower middle income were also excluded.

### Quality and risk of bias assessment

**Figure 1:**
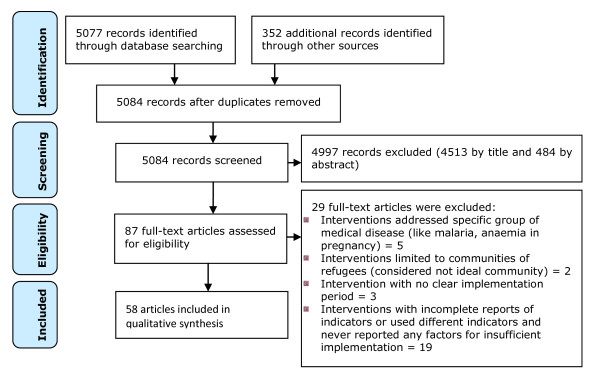
**PRISMA flow diagram of the process of identifying and including articles for the systematic review**.

The first author identified the articles, imported them into the EndNote X reference management software, removed the duplicates, examined the titles, then abstracts and retrieved the full text of relevant abstracts for further assessment (Figure 1). The first two authors independently assessed full-texts for inclusion in the review and completed the data extraction form for those that were eligible for inclusion. Uncertainties were resolved through discussions. Internal validation and generalizability of the included articles were carried out using a devised quality assessment tool (Additional file 2). The possibility of publication bias of RCTs and quasi-experimental designs with control groups was assessed using a funnel plot.

### Data collection and analysis

A designed eligibility form was used to document all full-text articles assessed for eligibility, the judgment reached whether a study was eligible and reasons in case of exclusion. A data extraction form was used to collect all necessary information from eligible articles. This form had six sections designed to collect information on (i) general information of the articles: name of reviewer, title, authors, year of publication, type of study (ii) general characteristics of the project: country involved, setting (whether rural, urban or both), sources of funds (whether local, external or both), total period of implementation, (iii) implemented interventions, (iv) outcome measures: results of interventions (v) underlying factors for implementation and (vi) quality and risk of bias. Indicators for outcome measures were changes in MMR and EmOC services indicators (also known as UN emergency obstetric care process indicators) i.e. case fatality rate (CFR), proportion of births in EmOC facilities, the met needs for EmOC and the caesarean section rate (CSR) before and after implementation of the intervention. Although the overtime changes of MMR may be confounded by a wide range of factors, it is generally acceptable as key health indicator used to evaluate the impacts of interventions for maternal care [8,9]. These indicators were preferred for this review because they are globally recommended for assessment, monitoring and evaluation of availability, utilization and quality of EmOC services [10]. All these items were entered initially into Excel software and then transferred to the Stata software for analysis. Supplementary information with a summary of characteristics of all studies included in this systematic review is available in the journal's website appendix (Additional file 3).

### Principal summary measures

The raw data were extracted from these articles followed by computation of the odds ratio and 95% confidence intervals for all indicators used in these studies. Meta-analysis was not carried out because of wide variations of packages, time intervals and indicators used for outcome measures of interventions.

## Results

Out of a total of 5084 articles published in English language resulting from the search, 87 full-text articles were assessed for eligibility. Of these only 58 qualified for systematic review and included 46 articles reporting maternal health interventional outcomes measured using MMR and/or EmOC services indicators. Of these 4 pairs of articles reported about the same programs and were thus merged to get a total of 42 interventional programs. Other articles included in this review were 12 interventional programs which used outcome measures other than MMR and EmOC services indicators but reported underlying factors for implementation in resource limited settings. The rest were excluded from the review with reasons (Figure 1).

**Figure 2:**
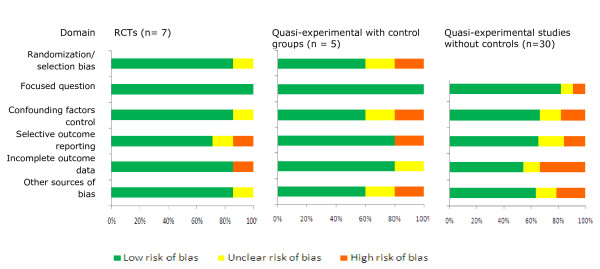
**Quality and risk of biases of included studies in the systematic review**.

**Figure 3:**
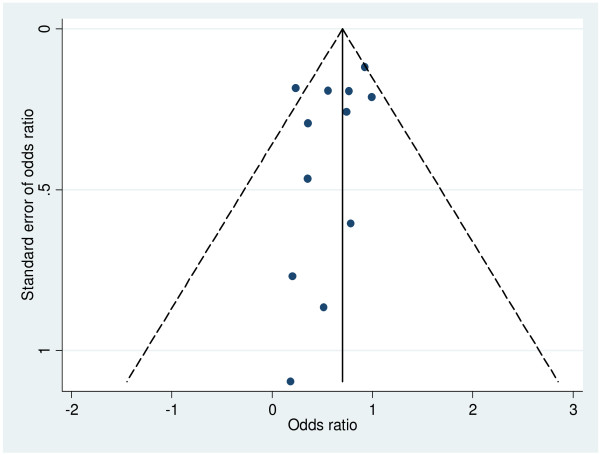
**Funnel plot with pseudo 95% confidence limits for RCT and cohort studies**.

The quality of RCTs and quasi-experimental designs with control groups were satisfactory as most of the information was from studies with low risk of bias (Figure 2). Expectedly there were more unclear and high risks of biases among the quasi-experimental designs without control groups than the former studies. Although the review team did not access the protocols for these studies (accessed only one [11]), no single study completed the set of EmOC services indicators and there was huge variability of the indicators across the studies suggesting the possibility of selective outcome reporting. The funnel plot for RCTs and quasi-experimental designs with control groups was asymmetrical suggesting increased possibility of high risk of publication bias especially for the small studies (Figure 3). However, this asymmetrical view could have been caused by other factors, such as differences in study qualities and heterogeneity. In view of this, the review team proceeded with further analysis although this selectivity posed a threat to the validity of the effects of the interventions.

### Implementation of the interventions

**Figure 4:**
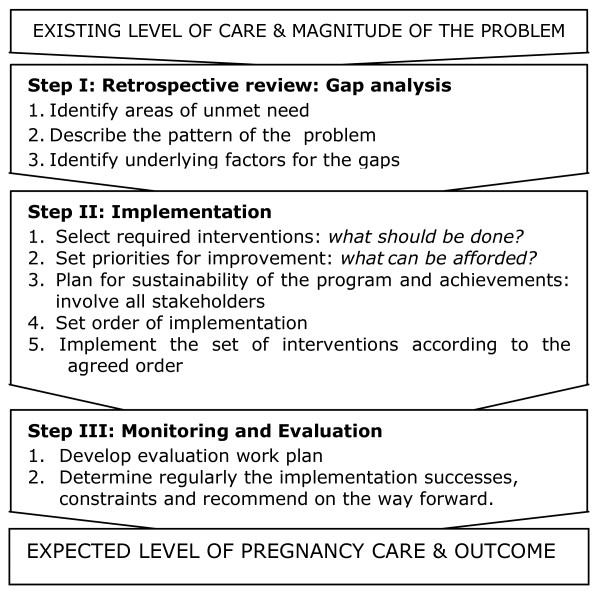
**Logical Systematic Implementation Framework for Maternal Health Interventions**.

Of all 42 interventional programs only 7 (17%) were randomized controlled trials, 5 (12%) quasi-experimental designs with control groups and the rest were quasi-experimental designs without control groups. From these interventional programs a list of interventions for maternal health was established (Table 1). The implementation processes of maternal health interventions followed almost the same simple logical framework with minimal variations (Figure 4). Mostly, the interventions were developed following descriptive retrospective reviews or needs assessments in the respective project areas which were conducted to identify the magnitude of the problem, the causes and underlying factors for the high ratios of maternal deaths. These pre-interventional studies were carried out in order to develop and implement more focused interventions. These activities were then followed by setting priorities, setting order of implementation of the interventions followed by monitoring and evaluation.

**Table 1:** Category, frequency and level of prevention of the interventions for maternal health extracted from the included programs

SN	Category of interventions	Level of prevention	Frequency/ percentage n = 54
**Community based interventions**			
1.	Community based Information, Education and Communication: Focus: *awareness of danger signs of pregnancy complications and birth preparedness and importance of health facility delivery services*	2,3	20 (37%)
2	Establishing community based funds for obstetric complications [loans and/or transport programs]:	2	10 (19%)
3	Training and/or linking traditional birth attendants to the health system: Focus: *Clean delivery and shorten delays for complications of pregnancy and childbirth*.	2,3	15 (28%)
4	Supplementing vitamin A or β carotene during pregnancy: To prevent infectious maternal morbidity and mortality	2	2 (4%)
**Health facility based interventions**			
5	Establishing/refurbishing blood banks and blood policies	3	11 (20%)
6	Training on CEmOC, placement and motivation of care providers	3	35 (65%)
7	Refurbishing/upgrading existing health facility infrastructure and equipment for obstetric care	3	28 (52%)
8	Improving supply of drugs, consumables and equipment for obstetric care	3	30 (56%)
9	Strengthening referral system and transport of patients	2,3	25 (46%)
10	Construction of new health facilities for CEmOC services	2	2 (4%)
11	Enabling policies and political commitment: Focus: To increase health facility utilization and accessibility of essential obstetric care services	1,2,3	10 (19%)
12	Establishment of revolving funds at the EmOC health facility	3	4 (7%)
13	Establishing family planning services	1	3 (6%)
14	Establishing maternity waiting homes	2	1 (2%)
15	Establishing mobile maternal health services, outreach and/or supportive supervision programs	2	7 (13%)
16	Improving and/or promoting antenatal care	2,3	6 (11%)

*Level 1: primary prevention (preventing pregnancy; 2: secondary prevention (preventing obstetric complications); 3: tertiary prevention (preventing death once obstetric complications have occurred).

Despite the diversity of country contexts and the multifaceted nature of maternal health and its determinants, the interventions overlapped considerably with each other across the programs. Attempts were made to classify the interventions according to level of prevention: interventions targeted at preventing pregnancy (primary), preventing obstetric complications (secondary) and preventing death once obstetric complications had occurred (tertiary prevention).

Training in comprehensive EmOC, placement and motivation of care providers, refurbishing existing health facility infrastructure and improving supply of essential drugs, consumables and equipment for obstetric care were the most frequent interventions integrated in 52% - 65% of the programs. Community-based information, education and communication (IEC) were involved in 37% (20) of the programs. The IEC addressed the danger signs of pregnancy complications, birth preparedness and complication readiness in an attempt to shorten delays through better awareness and promoting health facility deliveries.

The success of these programs was attributed to increased health facility deliveries, knowing when to reach out for assistance, and increased awareness and knowledge of the danger signs of pregnancy complications. Interventions which focused on linking traditional birth attendants (TBAs) with the health care system were implemented in various countries including Gambia, Honduras and some provinces in China. The training focused on creating TBAs' awareness of the importance and practice of clean delivery and early referral to the formal health care system with essential obstetric services to avoid delays in case of complications. Training of TBAs in countries where the community commonly utilized their services had strong impacts on maternal health outcomes only when it was supported by functioning referral systems and good working relationships with the formal health care systems.

All 22 interventional programs for maternal health from Sub Saharan Africa were small scale mainly confined to a hospital (district or state) and/or small surrounding communities. Countrywide reduction of MMR was reported in China, Bangladesh, Nepal, Honduras and Bolivia. In these countries the efforts targeted specific geographic areas with high ratios of maternal mortality with special attention to the most remote rural areas. The interventions included development of health facility accountability like health facility performance based funding (in some Chinese provinces), development of financing systems like community based funds and removing financial barriers to maternal health care services like the establishment of Maternal and Child National Insurance (Bolivia, China and Nepal). These policies were reported to increase women's access to skilled attendance.

### Impacts of the interventions

A wide range of indicators were used to assess the impacts of these maternal health care interventions. Depending on the type of intervention the most frequently used outcome (impact) indicators were MMR (67%) and EmOC services indicators (52%). Other indicators (not included in the analysis) were mean time from onset of complications/or admission to treatment, number of referred patients, utilization of obstetric and blood services, community awareness and knowledge on obstetric care. Of all RCTs only a study from Pakistan used at least one EmOC services indicator in addition to MMR. While only 2 (29%) RCTs reported statistically significant maternal mortality reduction, significant reduction was found in as high as 60% (3) of quasi-experimental designs with control groups (Table 2).

**Table 2:** The packages of interventions and their impacts for maternal health in resource limited countries

Country	Integrated interventions (from table 1)*	Time Interval (years)	Maternal deaths/live births		Odds ratio (95% CI)
				
			Intervention	Control	
**Randomized controlled trials**					
Zimbabwe [26]	16	2	6/9,394	5/6,138	0.78 (0.24 - 2.57)
Zimbabwe [27]	16	3	2/6,483	4/6,696	0.52 (0.09 - 2.82)
Pakistan [28]	3,15	2	27/10,093	34/9,432	0.74 (0.45 - 1.23)
India [29]	1	3	49/9,388	60/8,819	0.77 (0.52 - 1.12)
Nepal [30]	4	3.5	59/14,948	51/7,241	0.56 (0.37 - 0.84)
Nepal [31]	1	3	2/2,899	11/3,226	0.20 (0.04 - 0.91)
Ghana [11]	4	29 × 10^3 §^	138/39,601	148/39,234	0.92 (0.73 - 1.17)

**Quasi-experimental designs with control groups**					
Angola [32]	3,6	4	55/18,755	66/5,363	0.24 (0.16 - 0.34)
Gambia [33]	3, 6,9,15,16	3	1/769	5/714	0.43 (0.02 - 1.55)
Bangladesh [34]	3,6-9,15,16	3	6/4,424	20/5,206	0.35 (0.66 - 0.89)
Bangladesh^ф ^[35]	1, 3,6-9,13,16	4	41/10,890	50/13,169	0.99 (0.66 - 1.50)
Gambia [36]	3,8,9	7	11/405 to 13/1,236	4/267 to 7/727	0.36 (0.20 - 0.64)

**Quasi-experimental designs without control groups**			After intervention	Before intervention	
Egypt [1,37]	1,6-9,11	7	585/696,428	772/443,678	0.48 (0.43 - 0.54)
Senegal [38]	5-8,15	3	27/6,622	50/6,017	0.49 (0.30 - 0.80)
Pakistan [39,40]	3,9,11,13,15,16	5	34/52,982	48/55,454	0.74 (0.48 - 1.15)
Tanzania [41]	5,6,7,8	5	8/4,296	28/3,000	0.20 (0.09 - 0.44)
Peru [42]	1,6,7,9,15	4	2/3,119	9/3,002	0.21 (0.05 - 0.99)
Nigeria [43]	5,6,7,8	6	7/1208	47/2999	0.37 (0.17 - 0.81)
Nigeria [44,45]	1,2,6,7,8	6	7/815	44/861	0.16 (0.07 - 0.36)
Nigeria [46,47]	2,5-8,12	5	0/130	1/139	0.53 (0.02 - 16.02)
Bangladesh [48]†	1,3, 9,13,16	13	86/46,320	299/10^5^	0.62
Cameroon [49]†	6,9	10	60/10^5^	260/10^5^	0.23
China [1]†	1,3,5-9,11	16	61/10^5^	100/10^5^	0.61
China [50]†	1,3,6,9,11	3	114/10^5^	456/10^5^	0.25
Honduras [51]†	1,3,6-9,14	7	108/10^5^	182/10^5^	0.59
Bolivia [1,52]†	7-9,11	11	230/10^5^	390/10^5^	0.59
India [53]†	6-11	14	90/10^5^	380/10^5^	0.24
Nepal [54]†	1,2,5-8,11	10	281/10^5^	539/10^5^	0.52

Note: *The numbers given to the interventions correspond to those given in table 1; § Follow up years (the interventions were started in 2000 - 2008 but at different times across the clusters); ^ф ^Interventions were implemented at various intervals and we analyzed results from the last 4 years when a complete package was implemented. † Odds ratio was calculated from MMR before and after intervention due to incomplete raw data in the review articles.

Maternal mortality ratio was remarkably reduced by as high as 80% from 933 to 186/100,000 live births (OR = 0.20, 95% CI = 0.09 - 0.44) for only 6 years using exclusively locally available resources as reported from one of the regional hospitals in Tanzania (Table 3). Similarly, only 40% (6) of programs which used CFR to assess impact of their interventions reported statistically significant reduction. The four most successful interventional programs reduced CFR by over three quarters (i.e. 77% - 100%). The met need for EmOC was used as an outcome measure indicator in only 10 reports and the mean increase was as high as 149% (ranging from 24% to 444%) after a mean period of 5 years. Even the nine interventional programs which lacked community-based packages reported increased institutional deliveries by an average of 74% after a mean period of 6 years of health facility quality care improvement and development of enabling policies.

**Table 3:** Impacts of interventions for maternal health in quasi-experimental studies without control groups in resource limited countries

Country	Interventions (from table 1)*	Time Interval (years)	Odds ratio and 95% CI of EmOC services indicators		
			
			Births in EmOC facility	CFR	CSR
Tanzania [55]	5-8	4	3.00 (1.46 - 6.18)	0.66 (0.11 - 4.04)	1.51 (0.15 - 15.46)
Ethiopia [55]	6,7,8	4	1.26 (0.15 - 10.23)	0.47 (0.16 - 1.44)	1.64 (1.50 - 1.80)
Bangladesh [56]	1,6-8,11	6	2.36 (2.33 - 2.39)	0.04 (0.03 - 0.05)	2.00 (0.37 - 10.97)
Mali [57]	2,6,8,9	3	2.78 (2.70 - 2.86)	0.48 (0.32 - 0.73)	1.09 (0.94 - 1.25)
Peru [42]	1,6,7,9,15	4	0.96 (0.91 - 1.02)	0.06 (0.00 - 34.26)	2.53 (1.93 - 3.33)
Rwanda [58]	6,7,8	4	0.94 (0.88 - 0.99)	0.56 (0.26 - 1.20)	1.59 (1.41 - 1.60)
Mozambique [59]	6,7,8,9	4	3.91 (3.80 - 4.02)	0.54 (0.35 - 0.84)	-
Tanzania [60]	1	2	41 (10 - 171)	-	
India [53]	6-11	15	4.13 (1.99 - 8.55)	-	-
Nepal [54]	1,2,5-8,11	10	2.52 (1.04 - 6.11)	-	-
Vietnam [61]††	6-9,15	4	3.11 (2.53 - 3.82) 1.2 (0.99 - 1.46)	-	38 (2 - 636) 1.43 (1.02 - 1.99)
Pakistan [62]	3, 6,16	2.5	2.04 (1.83 - 2.28)	-	1.44 (1.09 - 1.91)
Mozambique [63]†	7,8,9,10	3	8.61 (8.04 - 9.23)	-	2.69 (2.54 - 2.85)
Senegal [38]	5-8,15	3	-	0.42 (0.09 - 1.84)	2.75 (0.27 - 27.72)
Tanzania [41]	5,6,7,8	5	-	0.32 (0.21 - 0.48)	3.83 (1.63 - 8.99)
Nigeria [43]	5,6,7,8	6	-	0.72 (0.31 - 1.66)	1.12 (1.07 - 1.17)
S Leone [64]	1,6-8,12	6	-	0.11 (0.03 - 0.36)	1.68 (1.50 - 1.87)
Nigeria [65]	2,6,7,8	4	-	0.89 (0.31 - 2.61)	17 (1 - 300)
Nigeria [44,45]	1,2,6,7,8	6	-	0.17 (0.07 - 0.39)	-
S Leone [66,67]	1,2,5,6,9	3	-	0.43 (0.08 - 2.21)	-
Nigeria [46,47]	2,5-8,12	5	-	0.17 (0.01 - 4.74)	-

Note: *The numbers given to the interventions correspond to those given in table 1; **CSR **= caesarean section rate; CFR = case fatality rate; † Only one part of the report is included here as the program had other sites and the data were not combined. †† *Had two interventional areas: a) Hai Lang district hospital and b) Hoang Hoa district hospital*.

### Factors for change

The problem of insufficient implementation of maternal health interventions was generally attributed to three main interlinking factors i.e. leadership and management, resources and end-user related factors. The leadership and management related factors included insufficient commitment of politicians and other key actors which led to insufficient funding of health systems, under utilization of available resources, lack of enabling policies for maternal health care, poor management, misplacement of priorities and lack of credibility, loyalty to the assignments, innovativeness and leadership skills. Other leadership and management related factors were social and political instabilities as reported in Angola and Sierra Leone. On the contrary, remarkable successes in reducing maternal mortality in China, Egypt, Honduras, Bangladesh and Bolivia (reported above) were attributed to strong political commitment, good leadership in reproductive health and presence of enabling policies.

Eleven articles linked insufficient implementation of maternal health interventions to lack of resources. These included limited national budgets for health care versus high costs required to scale up maternal health interventions. Under-funding of health systems resulted into lack of essential drugs, supplies and equipment, insufficient health facilities and qualified human resources as well as inefficient referral systems. The review indicated that more than three quarters (80%) of all included interventional programs were supported by the international donor community.

Four articles cited a pattern of end-user factors which were by large linked to cultural, low social and economic status and nutrition. Illiteracy and cultural factors like lack of autonomy in the decision-making process, early marriages and dietary practices during pregnancy affected nutrition, access to health services and community participation in implementation of maternal health interventions. In places where enabling policies did not exist as reported in Nigeria and Sierra Leone, poverty affected utilization of obstetric care services.

## Discussion

This study has revealed a heterogeneous picture for the nature, extent of integration of interventions and results. Generally, RCTs revealed insignificant impact of the interventions for maternal health. This could be explained partly by the fact that almost all (86%) RCTs studied the impact of single interventions and were implemented for shorter periods as opposed to quasi-experimental programs which integrated multiple interventions and for longer periods. These findings suggest that no single magic bullet intervention exists for reduction of maternal mortality and that all interventional programs should be integrated in nature in order to bring significant changes [12].

On the other hand, results from quasi-experimental studies constituted the body of evidence of the effectiveness of packages of interventions for maternal health across a wide range of settings especially when these are integrated. Findings from previous systematic reviews on maternal health interventions complement the evidences [5,13,14]. The degree of integration of these interventions depends on the context and the determinants of maternal health in the respective communities.

This study has indicated that most programs focused generally on promoting accessibility and utilization of health facility delivery services as well as improving the quality of care. All programs which most successfully reduced maternal mortality and remarkable EmOC indicators, had established functioning maternal health care systems with access to skilled birth attendants equipped with appropriate drugs, supplies and equipments and systems of referral to higher levels of care in the event of obstetric complications. Such successes could be explained by the fact that most maternal deaths occur during the period around giving birth, and that most life-threatening obstetric complications arise suddenly without warning signs and hence require appropriate and timely management [15]. The degrees of the impacts were associated with the type of packages of interventions, degree of integration, duration and efficiency of implementation, the presence of enabling policies and the magnitude of the problem before implementation. The logic dictates that the higher the CFR and MMR, the higher are the chances of attaining greater impacts in these indicators after implementation of effective interventions.

The degree of implementation of maternal health interventions and approaches were uneven across the regions. While the programs in Asia and North Africa tried to cover wide areas and range of interventions, those from Sub Saharan Africa were mostly confined to one or few health facilities and the surrounding communities. These findings can partly explain huge disparities in the progress achieved in reducing maternal mortalities among regions [8,16].

On the other hand, although wealth was one of the factors for maternal health care and hence MMR at the country level, it has been reported that, there is no straightforward relationship between the two, and that health system responsiveness has an explanatory power that is significantly superior to most other factors [17,18]. Huge disparities of MMRs exist even among countries with similar low economic powers. For instance, from 1980 to 2008 with the GNI per capita of 1,000 US$ Lesotho and Ivory Coast had increased MMR from 590/100,000 live births to 964 - 994/100,000 live births, while Bangladesh with even less income (520 US$) decreased MMR from 1329/100,000 live births to as low as 338/100,000 live births. Similarly, between 1980 and 2008 with GNI per capita between 200 US$ and 400 US$, Nepal successfully reduced MMR from 864/100,000 live births to 240/100,000 live births, but MMR remained almost unchanged in Sierra Leone (1240 to 1033/100,000 live births) and remarkably increased in Malawi from 632/100,00 live births to as high as 1140/100,000 live births [5,7].

Quite often lack of good quality leadership within the public service has been more closely linked to poor performance of the public sectors than to a lack of national resources [19,20]. The under-funding of the health systems in countries whose governments signed the Millennium Declaration in 2000, committed to reduce maternal mortality with 75% by 2015, and failure of most Sub Saharan African countries to meet the Abuja Commitment to allocate at least 15% of the national budget to the health sector reflect irresponsible commitments of these states' leaders. The problem of irresponsible commitments and lack of accountability can be tracked from the states' leaders and key health managers down to the care providers' level [21]. As a central factor, the health sectors in resource limited countries particularly in Sub Saharan Africa require more proactive leaders with stronger internal desire for change to turn commitments and promises into resources and actions in order to reduce maternal mortality. Leadership is a change agent, is all about getting things done and taking on the responsibility to influence others [22].

Based on these findings, a list of short and medium term strategies for action is proposed for countries with insufficient implementation of maternal health interventions. These include mobilizing political will and commitment, leadership development strategies, establishment of performance management and appraisal systems to enhance creativity and innovations in the domain of reproductive health, strengthen community participation, integrating non-governmental organizations into motherhood programs, sharing information within and among countries and empowering women with education, autonomy and economy [23-25].

### Potential limitations of the study

The increased possibility of publication and selective outcome reporting biases found in this study pose a great challenge, not only to the validity of the results of this review but also to investigators and editors of journals. Such biases indicate that only positive findings are reported and/or published. This may be misleading the global community with regards to policy and decision making on what interventions are effective for maternal health. The small number of articles reporting on the impacts of maternal health interventions using the stated indicators might have been contributed by language limitation (English). However, attempts were made to identify reports through a comprehensive literature search of relevant articles although some reports may have been missed. The fact that this review was limited to health sector interventions poses another limitation to this study.

## Conclusions

Success stories in the context of maternal mortality reduction exist around the world even in countries with limited resources. The compiled evidences in this article strongly suggest that it is possible to reduce maternal mortality in the circumstances of resource limited countries if the state and health sectors' key actors realize their commitments and responsibilities, embark upon the underlying factors and intensify efforts to implement the evidence-based interventions. These findings indicate that no single magic bullet intervention exists for reduction of maternal mortality and that all interventional programs should be integrated in nature in order to bring significant changes. This article presents a list of the evidenced-based packages of interventions, the context of leadership in healthcare today and proposes to the local governments, intergovernmental agencies, donors and the international community an evidence-based approach for effective change in the health sectors in resource limited countries in order to achieve the goal set for maternal survival.

## Abbreviations

AIDS: Acquired Immunodeficiency Syndrome; CI: Confidence interval; CFR: Case fatality rate; CSR: Caesarean section rate; EmOC: Emergency obstetric care; HIV: Human immunodeficiency virus; IEC: Information, education and communication; MMR: Maternal mortality ratio; OR: Odds ratio; PRISMA: Preferred Reporting Items for Systematic Reviews and Meta-Analyses; RCT: Randomized controlled trials; TBA: Traditional birth attendant.

## Competing interests

The authors declare that they have no competing interests.

## Authors' contributions

ASN participated in design of the study, searched the literature, reviewed the papers and drafted the manuscript. DPU participated in design of the study, reviewed the papers and contributed to writing of the manuscript. JvR participated in design of the study, advised on content and contributed to writing of the manuscript. All authors read and approved the final manuscript.

## Pre-publication history

The pre-publication history for this paper can be accessed here:

http://www.biomedcentral.com/1471-2393/11/30/prepub

## Supplementary Material

Additional file 1 **Detailed search strategies for each database involved in the systematic review**. The detailed search strategies specific for each database involved in the systematic review. Click here for file

Additional file 2 **Quality assessment tool for included articles**. A detailed structure of the tool used to assess the quality and risks of biases for included articles in the systematic review. Click here for file

Additional file 3 **Characteristics of all studies included in the systematic review: supplementary material**. This table is a supplementary material with a detailed account of the characteristics of all studies included in this systematic review. These characteristics include the study population, setting, sample size, intervention and outcomes. Click here for file
