# Supplementary material for: Maternal health interventions in resource limited countries: a systematic review of packages, impacts and factors for change
Source: BMC Pregnancy Childbirth. 2011 Apr 17;11:30. doi: 10.1186/1471-2393-11-30 (PMC3090370; doi:10.1186/1471-2393-11-30)
Supplement: Additional file 1 — Detailed search strategies for each database involved in the systematic review. The detailed search strategies specific for each database involved in the systematic review. [file 1471-2393-11-30-S1.DOC]

**Additional file 1. Detailed search strategies for each database involved in the systematic review.**

**PubMed**

((("maternal mortality"[MeSH Major Topic] AND reduction[Title]) OR interventions[Title]) OR (randomized[Title] AND controlled[Title] AND trials[Title])) AND ("humans"[MeSH Terms] AND "female"[MeSH Terms] AND (Randomized Controlled Trial[ptyp] OR Classical Article[ptyp] OR Controlled Clinical Trial[ptyp] OR Evaluation Studies[ptyp] OR Journal Article[ptyp]) AND English[lang] AND (medline[sb] OR jsubsetn[text] OR systematic[sb]) AND ("1987/01/01"[PDAT] : "2010/06/30"[PDAT]))Search limits: English, range 01.01.1987 to 30.06.2010

**Popline**

“maternal & mortality” [Title/ Keywords] AND “interventions / reduction/ randomized controlled trials” [Keywords]

**African Index Medicus**

1. maternal [Key Word] and mortality [Key Word]
2. mortality [Key Word] and reduction [Key Word]
3. mortality [Key Word] and interventions [Key Word]
4. maternal [Key Word] and care [Key Word]
