# Supplementary material for: Maternal health interventions in resource limited countries: a systematic review of packages, impacts and factors for change
Source: BMC Pregnancy Childbirth. 2011 Apr 17;11:30. doi: 10.1186/1471-2393-11-30 (PMC3090370; doi:10.1186/1471-2393-11-30)
Supplement: Additional file 2 — Quality assessment tool for included articles. A detailed structure of the tool used to assess the quality and risks of biases for included articles in the systematic review. [file 1471-2393-11-30-S2.DOC]

**Additional file 2: Quality assessment tool for included articles**

|  | **Domain** | **Scores*** | | |
| --- | --- | --- | --- | --- |
|  |  | **Yes** | **Can’t tell** | **No** |
| **Part I: Randomized controlled trials and cohort studies** | | | | |
|  | Addressed an appropriate and clearly focused question  *Consider if the question is focused in terms of:*   - *the population studied* - *the Intervention given* - *the outcomes pre-specified* |  |  |  |
|  | Similarity: intervention and control groups at the start   - *Units randomized* - *Free of selection bias across the groups* |  |  |  |
|  | Confounding factors adequately controlled in the design or analysis |  |  |  |
|  | Incomplete outcome data addressed   - *Each main outcome assessed in the standard, valid and reliable way* - *Attrition and exclusions reported and compared,* - *Reasons for both (attrition and exclusions) reported* |  |  |  |
|  | Free of selective outcome reporting   - *Outcomes of interest reported in the pre-specified way* |  |  |  |
|  | Other sources of bias |  |  |  |
| **Part II: Uncontrolled interventional studies** | | | | |
|  | Addressed an appropriate and clearly focused question  *Consider if the question is focused in terms of:*   - *the population studied* - *the Intervention given* - *the outcomes pre-specified* |  |  |  |
|  | Confounding factors adequately controlled in the design or analysis |  |  |  |
|  | Incomplete outcome data addressed   - *Each main outcome assessed in the standard, valid and reliable way* |  |  |  |
|  | Free of selective outcome reporting   - *Outcomes of interest reported in the pre-specified way* |  |  |  |
|  | Other sources of bias |  |  |  |

**Interpretations of score: Yes = low risk of bias; Can’t tell = unclear risk of bias and NO = high risk of bias*.
