# Supplementary material for: Maternal health interventions in resource limited countries: a systematic review of packages, impacts and factors for change
Source: BMC Pregnancy Childbirth. 2011 Apr 17;11:30. doi: 10.1186/1471-2393-11-30 (PMC3090370; doi:10.1186/1471-2393-11-30)
Supplement: Additional file 3 — Characteristics of all studies included in the systematic review: supplementary material. This table is a supplementary material with a detailed account of the characteristics of all studies included in this systematic review. These characteristics include the study population, setting, sample size, intervention and outcomes. [file 1471-2393-11-30-S3.DOCX]

**Characteristics of all studies included in the systematic review: supplementary material**

| **Study** | **Population** | **Sample size** | **Setting** | **Interventions** | **Outcome measures** |  |
| --- | --- | --- | --- | --- | --- | --- |
| Koblinsky M [1] | Women of reproductive age (15–45 years) | Women giving birth in China (number is not provided in the article) | Both rural and urban China | Community-based IEC; Training and linked TBA to the formal health system; training staff on EmOC; upgrading health infrastructure; improving essential equipment, supplies and drugs; strengthening referral system; enabling policies for maternal health care; establishing health insurance; family planning. | From 1980 to 1996 MMR ↓ from 100 to 61/10^5^ live births. |  |
| Kirkwood et al [11] | Women of reproductive age (15–45 years) | 207,781 women (104,484 randomly assigned to vitamin A supplementation and 103, 297 to placebo). | 7 districts in  Brong Ahafo Region in Ghana | Vitamin A supplementation | Maternal deaths in intervention area were 138/39,601 pregnancies vs 148/39,234 in the control. |  |
| Munjanja et al [26] | Women from families of middle to low income living in Harare, Zimbabwe | 15,994 women were recruited into  the study | Harare, Zimbabwe | Introduction of a new ANC model vs the traditional model. | Maternal deaths in intervention area were 6/9,394 vs 5/6,138 pregnancies in the control. |  |
| Majoko et al [27] | Women booking for ANC in the clinics in Gutu district in Zimbabwe | 13,517 women (new model = 6897 and standard n = 6620) | Gutu district in Zimbabwe | Use of a new (focused) ANC model vs the traditional model | Maternal deaths in intervention area were 2/6,483 vs 4/6,696 pregnancies in the control |  |
| Jokhio et al [28] | Pregnant women in Larkana district in Pakistan | 19,557 women (10,114 recruited in intervention areas and 9,443 in the  control areas) | Larkana, a rural district in Pakistan | Training and linked TBA to the formal health care system and outreach clinics for antenatal care | Maternal deaths in intervention area were 27/9,710 vs 34/8989 pregnancies in the control. |  |
| Tripathy et al [29] | Women aged  15–49 years residing in eastern India | 19,030 births during 3 years (2005–08). | Jharkhand and Orissa: two of the poorest states in eastern India. | Community-based IEC | Maternal deaths in intervention area ↓ from 16/2,347 to 7/3,110; and ↓ from 7/2,162 to 12/2,985 live births |  |

| **Study** | **Population** | **Sample size** | **Setting** | **Interventions** | **Outcome measures** |
| --- | --- | --- | --- | --- | --- |
| West et al [30] | Women of reproductive age in Sarlahi district | 44, 646 women | Rural southeast central plains of Nepal | Supplementation of vitamin A or â carotene vs placebo | Combined vitamin A or β-carotene ↓ mortality to 59/14,948 vs 51/7,241 pregnancies in the control (placebo). |
| Manandhar et al [31] | Married women of  reproductive age (15–49 years) in Nepal | 3,190 pregnancies  in intervention and 3524 in control areas. | Makwanpur district in Nepal’s central region | Community-based IEC | Maternal deaths were 2/2,899 in intervention vs 11/3,226 live births in the control area. |
| Schaider et al [32] | Pregnant women giving birth in the study area during the 4 years of study | 19,666 women giving birth | Rural area in Angola | TBA training and placement of skilled staff. | MMR after intervention was 55/18,755 live births vs 66/5,363 before. |
| Foord [33] | Pregnant women in the intervention and control areas | 1,516 pregnant women (Upper Baddibu 794 vs. 722 in Upper Baddibu the control) | West Kiang district, Gambia | TBA training; improving treatment and referral schemes and increasing numbers of visits to rural outreach areas | MMR in intervention area was 1/769 vs 5/714 live births in the control. |
| Fauveau et al [34] | Married women of  reproductive age (15–49 years) in Bangladesh | 21,824 women (control area = 11,564 and intervention area10,260) | Bangladesh: Matlab rural subdistrict of the Ganges-Meghna delta | Posting of midwives in project villages; upgrading of health facilities; strengthening of referral system and promotion of ANC services. | Between baseline 1984-1986 and 1987-1989 after intervention: MMR ↓ from 20/4,548 to 6/4,424 live births in intervention area and almost unchanged 20/5,177 to 20/5,206 live births in the Control area |
| Ronsmans et al [35] | Women of reproductive age (15–49 years) | Mothers giving birth to 24,059 live births in intervention [10,890] and control area [13,169] from 1990 - 1993 | Matlab rural subdistrict of the Ganges-Meghna delta in Bangladesh | Community-based IEC; deployment of community midwives; upgrading health facilities; strengthening referral system and promotion of ANC services. | MMR in intervention area was 41/10,890 and 50/13,169 in control area |
| Greenwood et al [36] | Pregnant women in Farafenni area of The Gambia | All women giving birth in the project areas. | Farafenni area of The Gambia | Training of TBA; improving supply of essential consumables, drugs and equipment as well as transport of patients | After 7 years of interventions, MMR ↓ from 11/405 to 13/1,236 in the intervention area vs 4/267 to 7/727 in control. |

| **Study** | **Population** | **Sample size** | **Setting** | **Interventions** | **Outcome measures** |
| --- | --- | --- | --- | --- | --- |
| Campbell et al [37];  Koblinsky M [1] | Women of reproductive age (15–49 years) in Egypt | All deliveries in the selected areas between 1992-1993 and 2000 | All governorates in Egypt | Improving supply of essential drugs, consumables, equipment and referral system; training of staff in EmOC; community based IEC, development of enabling policies for maternal health care | MMR after intervention was 585/696,428 vs 772/443,678 live births prior to intervention |
| Dumont et al [38] | Pregnant women in Dakar district, the capital of Senegal | 19,937 pregnant women admitted to the Roi Baudouin district hospital, for childbbirth and those referred to other facilities from 1998 – 2000 | Dakar district in Senegal | Establishing of blood services, training on EmOC and placement of skilled staff, renovation of infrastructure and improving supply of essential drugs, consumables and equipment. | Between baseline and after intervention CFR ↓ from 6% to 2.6%; and CSR ↑ from 6.2% to 6.7% |
| Bashir [39] | Pregnant women in the Faisalabad district in Pakistan | All women giving birth in the district | Faisalabad district in Pakistan | Training of TBA on danger signs; establishing emergency ambulance equipped with medicines and trained staff to rapidly transport women who develop complications | MMR ↓ from 10.1/10^3^ at baseline in 1977 to 1.9/ 10^3^ in 1987 after intervention. |
| Bashir et al [40] | Pregnant women in the Faisalabad district in Pakistan | 276,717 women gave live births from 1989 - 1993 | Faisalabad city in Pakistan | Training of TBA; intensive community education; improving ANC checkup; establishing free obstetric flying squad service, improving linkage between formal and informal health services and distribution of iron pills. | MMR ↓ from 48/55,454 at baseline in 1989 to 34/52,982 in 1993 after intervention |
| Mbaruku & Bergström [41] | Pregnant women in Kigoma urban and rural districts | 29,485 women giving birth from 1987 - 1991 | Kigoma districts (rural and urban) in Tanzania | Provision of essential drugs, supplies and repair of equipment; training of staff in EmOC; establishing blood transfusion services and improving referral system | Between 1984 and 1991 MMR ↓ from 28/3,000 live births to 8/4,296; and CFR ↓ from 4% to 0.6% |

| **Study** | **Population** | **Sample size** | **Setting** | **Interventions** | **Outcome measures** |
| --- | --- | --- | --- | --- | --- |
| Kayongo et al [42] | Pregnant women in northern provinces of Ayacucho, Peru | 11,904 women giving birth in EmOC facilities in the study areas. | Northern provinces  of Ayacucho in Peru | Improving infrastructure, facility setup and information systems; staff development and placement; supportive  Supervision; improving referral system and  the mobilization of civil society | At baseline (before 2001) and 2004: births in EmOC facilities remained almost the same (26% and 25% respectively); Met need for EmOC ↑ from 30% to 84%; CFR ↓ from 1.7% to 0.1%; CSR ↑ from 3.9% to 6.0%. |
| Ifenne et al [43] | Pregnant women in Zaria, Nigeria where 90% of the population live in rural area | 11,291 maternity admissions at Ahmadu Bello University Teaching Hospital in Zaria [1990 – 1995] | Zaria, Nigeria where 90% of the population live in rural area | Provision of essential drugs, supplies and equipment; renovation of maternity block and theatre room; training of staff in EmOC; establishing blood transfusion services and community based IEC. | In 1990 and 1995 CFR for obstetric complications ↓ from 14% to 11% |
| Oyesola et al [44] | Pregnant women in Kebbi State, Nigeria | 7,073 maternity admissions at Birnin Kebbi State hospital from 1990 - 1995 | Kebbi State (70% of the population live in rural area) in Nigeria | Provision of essential drugs, supplies and equipment; supportive supervision; training of staff on EmOC and establishing community based IEC. | At baseline in 1990 and 1995 after interventions number of maternal deaths and CFR for obstetric complications ↓ from 44 to 7 and from 22% to 5% respectively; institutional CSR ↑ from 12% to 16%; attended women with obstetric complications ↓ from 200 to 152. |
| Gummi et al [45] | Pregnant women in Kebbi State, Nigeria | 10,135 maternity admissions in 3 Kebbi State project facilities from 1990 - 1995 | Kebbi State, Nigeria with 70% of the population living in rural area | Renovation of health facility infrastructure for maternal health care; deployment and training of staff on EmOC; referral system improvement and community based IEC | Births in EmOC facility slightly ↓ from 861 (1990) to 815 (1995) at Birnin Kebbi State hospital, institutional births ↑ from 162 in 1990 to 682 in 1995 at Jega Health Centre |
| Ande et al [46] | Women in reproductive age in Ekpoma district in Nigeria | 1,592 maternity admissions at Ekpoma district hospital from 1990 - 1995 | Ekpoma - a rural district in Nigeria | Provision of essential drugs, supplies and equipment; renovation of maternity block and theatre room; training staff in EmOC; establishing blood transfusion services and revolving fund | Institutional CSR ↑ from 0 in 1990-1991 to between 5% - 10% in 1992 – 1995; utilization of EmOC and laboratory services; CFR ↓ from 14% to 0 |

| **Study** | **Population** | **Sample size** | **Setting** | **Interventions** | **Outcome measures** |
| --- | --- | --- | --- | --- | --- |
| Chiwuzie et al [47] | Pregnant women in reproductive age in Ekpoma district in Nigeria | 2,273 one-time contributors to the fund 1995 | Ekpoma rural district in northen Nigeria | Establishing loan fund for women with obstetric emergencies; establishing supportive supervision and transport system. | 456 women requested for loan in 1995. |
| Chaudhury & Chowdhury [48] | Women reproductive age in Bangladesh | 684,328 women reproductive age in the project areas. | 592 villages in 11 districts in Bangladesh | Training of TBA to handle normal deliveries; provision of family planning services; promotion of ANC services and nutrition education; and village-level social auditing of maternal and perinatal deaths | MMR ↓ from 299 in 1993-1997  to 86/10^5^ live births in 2002-2005 |
| Nasah et al [49] | Pregnant women in Yaounde in Cameroon | All maternity admissions at Central Maternity (CM) and the  University Hospital Centre (UHC) | Yaounde, the  capital city in Cameroon | Establishing family planning services; deployment of personnel and restricted resources and training staff in EmOC. | From I978- I987 MMR ↓ from 200 to 60/ 10^3^ live births in CM and maintained at O-O.84 per 10^3^ at UHC |
| Xu [50] | Pregnant women in rural China | All women giving birth in the local township, county and maternity hospitals | Miyun County (outside Beijing), China | Training of staff in EmOC and TBAs; community education; provision of easier access to EmOC services; establishing obstetric rescue teams; strengthening referrals and improving MCH services | MMR ↓ from 456 to 114/10^5^ live births from 1985-88 |
| Danel [51] | Pregnant women in rural Honduras | All women giving birth in Honduras | Rural areas with highest MMR in Honduras | Training and deployment of clinical staff and community health workers including TBA and linking them to the health care system; strengthening referral; improving infrastructure for EmOC and maternity waiting homes | MMR ↓ from 182 to 108/10^5^ live births |
| Koblinsky M [1]; Kwast [52] | Pregnant women in Bolivia | All women giving birth in Bolivia from 1989 and 2000 | Bolivia | Provision of essential drugs, supplies, equipment, family  planning services; training of  community birth attendants; strengthening referral system; reduction of costs for emergency admissions | MMR ↓ from 390 at baseline in 1989 to 230/10^5^ live births in 2000 |

| **Study** | **Population** | **Sample size** | **Setting** | **Interventions** | **Outcome measures** |
| --- | --- | --- | --- | --- | --- |
| Padmanaban et al [53] | Women of reproductive age in a state of Tamil Nadu in India. | All women giving birth in the study area. | A state of Tamil Nadu in India | Training of staff in EmOC, placement of staff; renovation of health facilities; improving essential supplies and drugs; strengthening referral system and development of enabling policies. | From baseline in 1996 to 2007 institutional deliveries ↑ from 65% to 98%; MMR ↓ from 380 (1993) to 90/10^5^ live births (2007) |
| Barker et al [54] | Pregnant women in the project districts in Nepal | All women giving birth in the project districts | 25 project districts in Nepal | Staff training; improving infrastructure and equipment; promoting ANC; establishing community emergency funds and transport schemes; policy development and planning including increasing equity and access. | From baseline in 1997 to 2006 births in EmOC facilities ↑ from 4% -11%; Met need ↑ from 7.3% - 18.5%; CSR ↑ from 3.6% - 28%; CFR ↓ from 0.5% - 0.4; MMR ↓ from 539 to 281/10^5^ live births. |
| Kayongo et al [55] | Pregnant women in the study areas in Rwanda, Tanzania and Ethiopia | All women giving birth in EmOC facilities in the country study areas. | 10 rural hospitals in Rwanda, Tanzania and Ethiopia | Provision of equipment, essential supplies and  drugs; establishing blood transfusion services, training of staff in EmOC, strengthening information systems to monitor change and identify gaps in quality; infrastructure upgrades—renovations and equipment | Births in EmOC facilities ↑ from 13% to 18% (TZ), 1.6% to 1.8% (Ethiopia); Met need ↑ from 9% to 21% (TZ) and 2% - 5% (Ethiopia); CSR ↑ from 1% - 2% (TZ) and 0.2% - 0.3% (Ethiopia); CFR ↓ from 3%-2.4% (TZ) and 10.4% - 5.2% (Ethiopia) from baseline to the last year of the study. * Rwanda’s actual baseline data were unavailable |
| Islam et al [56] | Pregnant women in Bangladesh | All institutional deliveries (no raw data) taking place within the study period | *Khulna division in Bangladesh*  10 district hospitals and 19 upazila (sub-district) health complexes | Community based IEC; renovation of the facilities; developing human resources; supply of necessary equipment and logistics; and strengthening the management information system | From baseline before 1997 to 2002 births in EmOC facilities ↑ from 5.3% -11.7%; Met need ↑ from 4922/ 46,076 to 12,277/46,154; CSR ↑ from 1600/307,174 to 4099/315307; CFR ↓ from 3.3% to 1.6% |
| Foumier et al [57] | Pregnant women in west Mali | 51,384 institutional deliveries taking place within the study period | 6 rural health districts in Kayes region in the west of Mali | Establishing community based funds for EmOC; training of staff in EmOC; improving referral system, supply of drugs, consumables and equipment. | Births in EmOC facility doubled from 9,871/ 52,046 to 19,235/ 48,846; CSR from 112/475 to 383/913; CFR ↓ from 48/475 to 47/913 |

| **Study** | **Population** | **Sample size** | **Setting** | **Interventions** | **Outcome measures** |
| --- | --- | --- | --- | --- | --- |
| Kayongo et al [58] | Pregnant women in Gitarama province  in Rwanda | 10,308 institutional deliveries taking place at Kabgayi regional hospital from 2001 to 2004 | Gitarama province  in Rwanda | Hospital renovations, provision of essential equipment, training of staff in EmOC and improving management systems | Met need ↑ from 16% to 25%, CSR ↑ from 1.9% to 3.2%); CFR ↓ from 2.2% to 1.2% in 2001 at the start of the project and 2004 respectively. |
| Santos et al [59] | Pregnant women in Sofala province in Mozambique | 110,171 women giving birth in the project area from 2002 - 2005 | 23 project health facilities in Sofala province in Mozambique | Improving infrastructure, referral system, supply of drugs, consumables and equipment and training of staff in EmOC. | From a baseline in 1999 to 2005 after intervention: Births in facilities ↑ from 24,766/65,427 to 28,671/72,752; CFR ↓ from 32/1108 to 57/3586; CSR ↑ from 703/65,427 to 1,277/72,752 |
| Mushi et al [60] | Pregnant women in Mtwara rural district in Tanzania | 512 deliveries which occurring between October 2004 and November 2006 | Mtwara rural district in Tanzania | Promoting early and complete ANC visits and delivery with a  skilled attendant | Births in EmOC facility ↑ from 86/158 at baseline to 255/512 after intervention |
| Otchere & Binh [61] | Pregnant women in Vietnam | All women giving birth in Hai Lang and Hoang Hoa  districts in Vietnam | Thanh Hoa province in the north and Quang Tri province in central Vietnam | Training of staff in EmOC; upgrading operating theaters, labor and delivery rooms, postpartum wards and  laboratories; provision of essential equipment and supplies; strengthening referral system and supportive supervision, community based IEC. | Hai Lang Hospital  Births in EmOC facility ↑ from 154/1200 at baseline (1999) to 398/1268 at the end of 2004; Met need ↑ from 29/180 to 166/190; CSR ↑ from 0 to 20/1268.  Hoang Hoa hospital  Births in EmOC facility ↑ from 231/1241 in 1999 to 283/1311 in 2004; Met need ↑ from 32/186 at baseline to 107/197 in 2004); CSR ↑ from 61/1241 to 90/1311; CFR for the two hospitals remarkably remained at zero. |
| Lodhi et al [62] | Rural pregnant women in Kasur district | 3462 women giving birth in EmOC facilities from 2000 to 2002 | Both semi-urban and rural settings within the district of Kasur in Lahore province in Pakistan | Reinforcing regular ANC clinics; training staff in EmOC, anesthesia and laboratory; training and linking TBA to the health system | Births in EmOC facility ↑ from 442/ 6,942 to 1,694/13,883; Met need ↑ from 379/1041 to 1091/2082; CSR ↑ from 62/6,942 to 310/13,883 |

| **Study** | **Population** | **Sample size** | **Setting** | **Interventions** | **Outcome measures** |
| --- | --- | --- | --- | --- | --- |
| Jamisse et al [63] | Pregnant women in Maputo and Sofala provinces. | 272,247 women giving birth in EmOC facilities  within the study period in Maputo *(the number of deliveries in Sofala province was not provided)* | *Maputo and*  Sofala provinces in Mozambique | Training of staff in EmOC; provision of essential equipment, supplies nad drugs; upgrading and increasing the number  of health facilities providing basic and comprehensive EmOC; establishing of radio communication and rapid transportation of patients requiring comprehensive  EmOC | *Maputo province (1998–2001)*  CSR slightly ↑ from 3952/69,495  to 4,717/74,845; institutional deliveries 62,646/69,495 to 73,907/74,845  *Sofala province (2000 2002)*  CSR remained 1%; Met need for EmOC ↑ from 6.3% to 11.5%; births in EmOC facilities ↑ from 12% to 25%; CFR ↓ in basic EmOC units from 4.7% to 2.4% and from 4.1% to 3.1% CEmOC. |
| Leigh et al [64] | Pregnant women in Makeni district | 1480 maternity admissions at Makeni district hospital [1989 to 1995] | Makeni district in Sierra Leone | Deployment and training of staff on EmOC; establishing blood transfusion services and revolving fund; provision of essential drugs and supplies; and community-based IEC | From 1990 to 1995 number of births in EmOC facility ↑ from 42 to 84; number of maternal deaths ↓ from 10 to 5 and CFR ↓ from 32% to 5% |
| Olukoya et al [65] | Pregnant women in Ota town (mixed urban and rural populations) | 1437 maternity admissions at Ogun State hospital [1992 to 1995] | Ogun State in Nigeria | Improving electricity supply, provision of essential drugs and supplies and equipment, training of staff in EmOC services | CFR for major obstetric complications remained unchanged [6.6% in 1995 vs 7.3% in 1992] |
| Sengeh et al [66] | Pregnant women in northern part of Bo district in Sierra Leone. | All maternity admissions at Bo district hospital from 1992 to 1995 | Northern part of Bo district [with a population of about 350,000 people] in Sierra Leone. | Establishing blood transfusion services; provision of essential equipment, drugs and supplies; training of staff in EmOC and provision of community-based IEC. | Number of maternal deaths and CFR ↓ from 28 to 17 and from 13% to 10% in 1992 and 1995 respectively |
| Samai & Sengeh [67] | Pregnant women in northern part of Bo district in Sierra Leone. | All maternity admissions at Bo district hospital from 1992 to 1995 | Northern part of Bo district [with a population of about 350,000 people] in Sierra Leone. | Improving patients’ transport and installation of radio calls in primary health units; community-based IEC. | Women with obstetric complications attended in BDH ↑ from 0.9 to 2.6 per month; CFR of women from the project areas ↓ from 20% to 10% before (1991-1992) and after intervention (1992-1993). |

**Note:**

1. ANC = antenatal care; IEC = Information, education and counseling; TZ = Tanzania; EmOC = emergency obstetric care; CSR = Caesarean section rate; CFR = case fatality rate; MMR = maternal mortality ratio/ rate; TBA = traditional birth attendants;
2. The number of references in this table correspond to those in table 2 and 3 as well as the list of references in the article.
3. Definition of terms: Proportion of births in EmOC facilities: number of births in EmOC (basic and comprehensive) facilities divide by expected number of births in a given area; Met need for EmOC: proportion of women with obstetric complications treated in the EmOC facilities; CSR: proportion of all abdominal births in a given area; CFR: proportion of women admitted with obstetric complications in the EmOC facilities who die.
